# The Natural Moisture of ABS Filament and Its Influence on the Quality of FFF Products

**DOI:** 10.3390/ma16030938

**Published:** 2023-01-19

**Authors:** Adam Hamrol, Błażej Góralski, Radosław Wichniarek, Wiesław Kuczko

**Affiliations:** Faculty of Mechanical Engineering, Poznan University of Technology, Piotrowo 3, 60-138 Poznan, Poland

**Keywords:** additive manufacturing, fused filament fabrication, ABS, ambient humidity, filament moisture, tensile strength, surface quality

## Abstract

The article presents the results of research on the influence of the natural moisture of a filament made of acrylonitrile–butadiene–styrene terpolymer (ABS) on the mechanical properties and quality of products fabricated with fused filament fabrication (FFF). The concept of the natural moisture of the filament was defined, and the range of its variability was identified in reference to the range of the natural ambient humidity. It is shown that a change in the ambient humidity by 10% resulted in a change in filament moisture by about 0.05%. The results of the research on the moisture variability of an ABS filament stored in a package, an airtight container, or a container with a moisture absorber are also discussed. The last part of the article presents the results of the research on the impact of the moisture of the filament in its natural range of variability on select mechanical properties of filaments and products made using FFT. It is shown that this impact was significant and had a value of 1 MPa on 0.1% filament moisture.

## 1. Introduction

Fused filament fabrication (FFF) is one of the most widely used additive manufacturing technologies. Devices and materials used in FFF are relatively cheap, and the technology itself is easy to learn when compared to selective laser sintering, for example. Therefore, FFF is characterized by a low implementation threshold in terms of technology and economy. This is one of the reasons why FFF is used by small companies for the production of innovative products [1,2,3], as well as for the production of product prototypes [4,5].

The material used in FFF is capable of transformation under the influence of heat from a solid state to a viscous state and vice versa. Most often, it is used in the form of a thin fiber (1.75 or 3 mm in diameter) called a filament [6,7].

Filament properties—important in the context of the quality of FFF products—are determined, among other methods, by the glass transition temperature, material shrinkage, temperature resistance, mechanical strength, flexibility, solubility, approval for contact with food, and emission of harmful vapors [8]. These properties depend on the type of polymer the filament is made of and the dyes and fillers used [9,10,11,12].

The properties of the filament, its behavior in processing, and the quality of manufactured products are largely influenced by the filament moisture, which is a measure of the amount of water contained in the filament. This is determined as the ratio of the weight gain resulting from the water absorbed by the material to the weight of this material after drying to the residual moisture.

Polymer materials are characterized by a different ability, called hygroscopicity, to absorb and retain water from the environment. Due to the location of accumulation of water in the polymer, the surface, capillary, and cellular moisture are distinguished. [13]. Surface moisture occurs only on the outer surface of the filament. Capillary moisture accumulates in various types of pores, crevices, and irregularities on the surface of the material. Cellular moisture is water contained deep within the structure of the filament.

In non-hygroscopic materials, such as polypropylene and polystyrene, moisture settles only on the outer surface. In hygroscopic materials, such as acrylonitrile–butadiene–styrene (ABS) and polyamide, water particles are absorbed into the material. The amount of water absorbed by the material depends on the duration of exposure in the natural environment and the degree of its moisture.

The process of moisture penetration into the filament in the form of water vapor contained in the air is called moisture sorption, and it is divided into:

- Adsorption and adhesion of water molecules to the surface of the material;
- Absorption and penetration of water molecules into the volume of the material.

Moisture absorption is diffusive and consists in the movement of moisture particles into the structure of the material. The spaces for diffusible water molecules are the voids between the polymer chains. If the polymer does not react with water, its molecules remain unbound with the polymer and can move freely. In such cases, it is possible to simply remove the moisture from the material by drying it. In the cases of polymers with high affinity with water and those containing polar groups, the formation of hydrogen bonds and the immobilization of water molecules occurs, which results in the swelling of the material [14,15,16].

It is assumed that the surface layer of the material reaches saturation immediately, and the absorption coefficients depend on the rate of diffusion of the absorbed substance from the surface to the deeper structures of the material [17,18,19].

During drying, the phenomenon of desorption occurs (i.e., the removal of excess water from the polymer). The intensity of the release of particles into the environment depends on the amount of water absorbed, the bonding strength of water with the polymer, and the depth of water penetration. For this reason, hygroscopic materials, as opposed to non-hygroscopic materials, require long-term drying in order to obtain the desired level of desorption.

The problem of filament moisture influence on the quality of printed products is of interest to many research centers. Zaldivar et al. proved [20], for example, that the moisture level of polyetherimide (PEI) material supplied by a manufacturer ranges from 0.12–0.18%. Exposing it to humidity in special climatic chambers increased the moisture of the filament to a maximum value of 0.8%. Zaldivar also presented studies showing the negative effect of filament moisture on the mechanical properties of PEI products. He showed that in the moisture range of 0–0.16%, the deformation capacity of products was 150% higher in comparison to samples of products with a moisture content of 0.4–0.8%.

Valerga et al. demonstrated that the moisture of a polylactide (PLA) filament influenced the glass transition temperature and the value of the maximum tensile stress of the filament. They also found that dry filament conditioned in an environment with humidity of 16% makes possible a product with greater strength but is more brittle than dry filament conditioned with humidity of 50% [9].

Halidi and his team stated that in the case of filament made of ABS, the absorption of moisture reached a certain maximum point, and then remained constant. The moisture in the filament affected the filament’s stickiness and ability to flow. They also observed that an increase in the moisture caused an increase in the filament diameter, but did not limit the flow inside the nozzle [21]

Research conducted by Wichniarek showed that in natural conditions, the moisture of ABS material may vary from 0.1–0.6%. It was also found that the influence of moisture in the material had a significant impact on the course of the manufacturing process and the properties of the manufactured products. The tensile static strength and the modulus of linear elasticity decreased [22] with an increase in filament moisture.

Similar conclusions were presented by Wittbrodt and Pearce. They proved that the humidity in the environment or in the storage chamber of a PLA filament can cause changes in the behavior of the material during printing and noticeable changes in the mechanical properties [23].

Banjo et al. conducted a flexural strength test of dry-conditioned PLA materials conditioned for 14 days in deionized water at 70 °C and 21 °C. The tests showed a change in humidity in the range of 0.7% to 0.9%. According to the authors, this change was insignificant, because PLA has lower hygroscopic properties compared to other materials (e.g., nylon), thanks to which it absorbs smaller amounts of water from the environment [18].

Filament additives in the form of fillers and reinforcing fibers play an important role in the absorption of moisture. Additives in the form of organic particles and natural fibers absorb moisture better than the base polymer. The effect of additives on the level of water absorption in the case of nylon reinforced with carbon fibers depends on the volume fraction of the base polymer [24]. Research carried out by Kariz et al. showed that the addition of wood fibers to the PLA filament caused a significant increase in hygroscopicity. Filaments with a 10% addition of fibers, stored in a humid environment, had a moisture content about three times higher than filaments without additives stored under the same conditions. The addition of fibers at the level of about 30% caused an almost fivefold increase in the moisture content of the filament [25].

The papers [26,27,28,29] investigated the tensile strength, elongation, and creep phenomenon of samples made of PLA, PC, ABS, nylon, and composites of filaments with different moisture contents. All of them showed a negative influence of humidity on the above-mentioned mechanical properties.

The results of selected tests, showing the scope of changes in the moisture of the filament and the mechanical properties of products made of them, are summarized in Table 1.

Although the influence of filament moisture on its properties and the course and results of FFT processes is the subject of many scientific papers, it should be recognized that this topic has not been fully researched and described. First of all, there is no clear information on the scope of the variability of the filament humidity during its storage in natural conditions. In tests described in the literature, a certain level of moisture is usually obtained by force (e.g., by soaking it in water). As a result, it is not clearly explained to what extent changes in the filament moisture within the natural range of its variability affect the mechanical properties and other quality properties of FFF printouts. The study of these relationships was the motivation to carry out the research and analysis presented in this article, which will fill a significant cognitive gap. 

This research is also of great practical importance. Observations have shown that, in practice, the moisture of the filament is ignored by the FFF process operators. Usually, it is only during the printing process that they notice that the filament moisture is too high. It manifests itself, for example, by the escape of water vapor from the nozzle and audible mini explosions of trapped water, as well as clear defects in the product surface. More profound knowledge about the influence of ambient humidity on the humidity of the filament will allow users to take action regarding improving the storage or the necessity of drying the filament.

## 2. Methods 

### 2.1. Definition of Natural and Special Filament Moisture

The moisture of the filament (FM) can be expressed as:

*FM* = *f*(*MH, AH, AT, AP, SC, t*) (1)

where:

− *MH*—material hygroscopicity (depends on the type of polymer and additions to the polymer, such as stabilizers, oxidants, etc.);
− *AH*—ambient humidity;
− *AT*—ambient temperature;
− *AP*—atmospheric pressure;
− *SC*—special factors (e.g., air movements, method of storage);
− *t*—time of the impact of the humidity from the environment on the moisture of the filament.

Depending on how these factors affect the process of water absorption by the filament, three ranges of filament moisture can be distinguished:

− Residual;
− Natural;
− Special.

Residual moisture is the moisture in the filament after the drying process until the so-called dew point [31].

The terms natural and special moisture are understood by analogy with the concept of natural and special process variability used in statistical process control [32,33]. Natural variability is the result of the impact on the process of the natural causes that arise because of natural circumstances. Natural causes are also called common causes, noise, non-assignable, or random causes. These causes are an inherent part of the process and cannot be practically eliminated without using special, usually expensive sources. Special variability is the opposite of natural variability. It is a result of special causes that arise because of the special circumstances that are not an inherent part of the process. Special causes are also referred to as assignable causes. 

Natural moisture is obtained in the filament during the storage process under natural environmental conditions. The natural conditions of the environment are determined by the air temperature, ambient humidity, and atmospheric pressure within the range of their variability occurring during filament storage under production conditions. The following ranges of the variability of natural conditions were assumed:

− Air temperature: 18–30 °C;
− Ambient humidity: 30–80%;
− Atmospheric pressure: 980–1040 hPa.

Depending on the environmental conditions, the natural moisture of the filament changes with time; the scope of these changes can be called natural moisture variability. These changes cannot be eliminated because it would require significant expenditure, such as maintaining constant and controlled temperature and humidity in rooms or in containers for filament storage and special supervision of production processes.

Special moisture is acquired by the filament in the storage process as a result of the action of special causes. They appear, for example, in the form of an uncommon source of moisture or a combination of uncommon high ambient temperature and humidity. A special cause can also arise due to the storage of the filament in a container with a moisture absorber that absorbs the moisture contained in the filament material.

**Figure 1:**
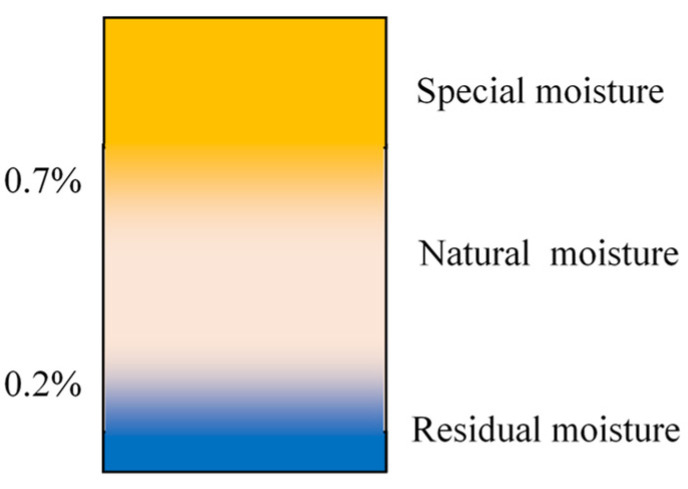
Ranges of filament moisture.

The boundaries between residual, natural, and special moisture are not sharp, as shown in Figure 1.

### 2.2. Goals

The aim of the research was to determine:

− The range of the ABS filament moisture stored in different ambient humidity levels (the range of natural moisture), with the other variables in Equation (1) assumed to be constant;
− The impact of ABS filament natural moisture variability on the mechanical properties of the filament;
− The impact of ABS filament natural moisture variability on the mechanical properties of the samples produced with the FFF method.

To achieve these goals, the following scope of research was planned:

1. Determination of the range of variability of the filament moisture:
  − Provided by various manufacturers;
  − Stored in normal environmental conditions, within the scope of the natural variability of the ambient humidity (with only slight changes in temperature and pressure);
  − Stored in a vacuum container or in a container with a desiccant;
2. Testing the mechanical properties of the filament depending on its moisture;
3. Testing the mechanical properties of samples fabricated by FFF depending on the filament moisture.

### 2.3. Materials 

A filament made of ABS (Table 2) was selected for the research because it is one of the most commonly used materials in FFF due to its properties and the possibility of further mechanical processing [34,35]. ABS is used primarily for making prototypes of products, and for equipment housings, the production of instruments, spare parts, and much more [36,37,38].

### 2.4. Measurements Methods

**Figure 2:**
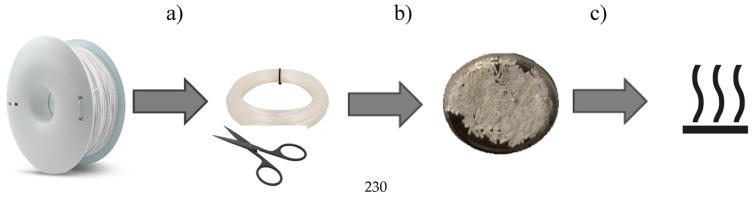
Scheme of determining the moisture contents of filament samples: (**a**) cutting off the samples weighing about 10g, (**b**) cutting the samples into sections, (**c**) drying.

The filament moisture was measured on a RADWAG (Radom, Poland) balance dryer, type MA50/1.R, from filament samples with a length of about 10 mm (weight ca. 10 g). A diagram of the filament moisture test process is shown in Figure 2. The drying temperature was set to 80 °C, while the drying time was set to 90 min. [40].

**Figure 3:**
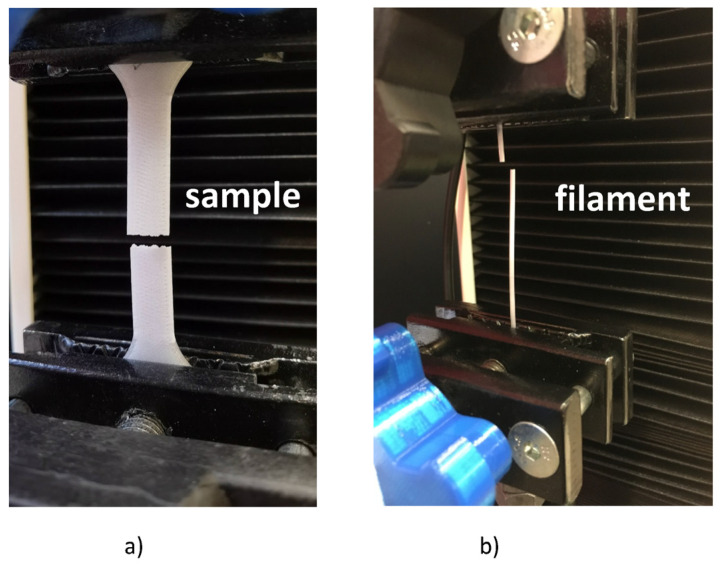
The strength tests of the filament and the printed samples: (**a**) strength tests of printed samples, (**b**) strength tests of the filament with a diameter of 1.75 mm.

The tensile strength tests of the filament and the produced samples were carried out on the SUNPOC (Guiyang, China) WDW-5D measuring machine (Figure 3). The fiber samples had the same dimensions as the dried samples. The geometry of the printed samples was developed on the basis of the PN-EN ISO 527:1998 [41] standard, which defines the conditions for tensile testing for plastics. The pattern was based on the type B1 of a sample called a paddle.

## 3. Results and Discussion

### 3.1. Commercial Filament Humidity

**Figure 4:**
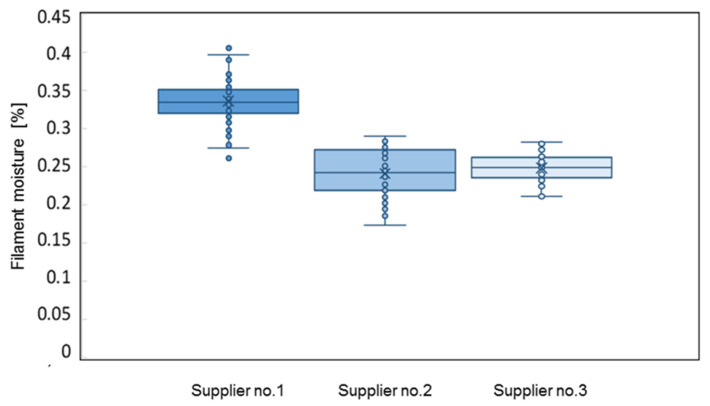
Moisture contents of filaments provided by various suppliers.

The moisture contents of ABS commercial filaments purchased from three suppliers were tested. The filaments from all manufacturers were delivered on spools in the original packaging. The moisture test was performed immediately after opening a package, at an ambient humidity of 52–54%. The measurement results are shown in Figure 4.

The filaments showed moisture differences ranging from 0.18 to 0.40%. It can be assumed that these differences were the result of differences in the production processes used by the manufacturers (e.g., proportions of ingredients), the method of packaging, protection against moisture, and delivery to the end customer.

### 3.2. Filament Moisture from One Supplier

**Figure 5:**
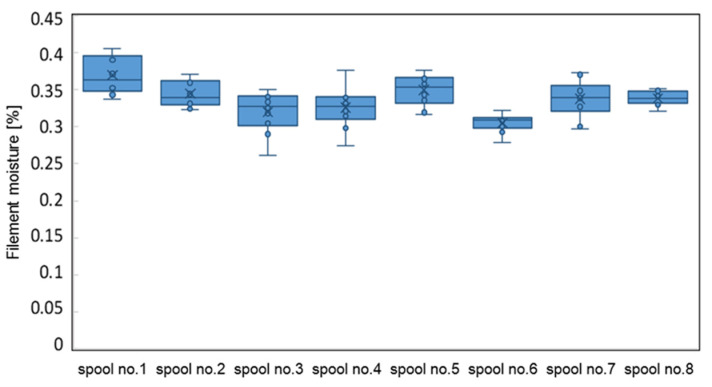
Differences in the moisture of the filaments from one manufacturer.

The moisture measurement was carried out on filaments taken from six different spools from one supplier, immediately after opening the package. Samples for the moisture testing were taken from various places on the spool (top, middle, and inner layers). The obtained results are shown in Figure 5.

The results show that the filament supplied by one manufacturer had a moisture content variation range of 0.23–0.40%. The explanation for this variability may be different conditions of collecting or releasing moisture, depending on the “position” of the filament on the spool (outside/inside), as well as the heterogeneity of the material itself.

### 3.3. Changes in the Filament Moisture Depending on the Humidity of the Environment

**Figure 6:**
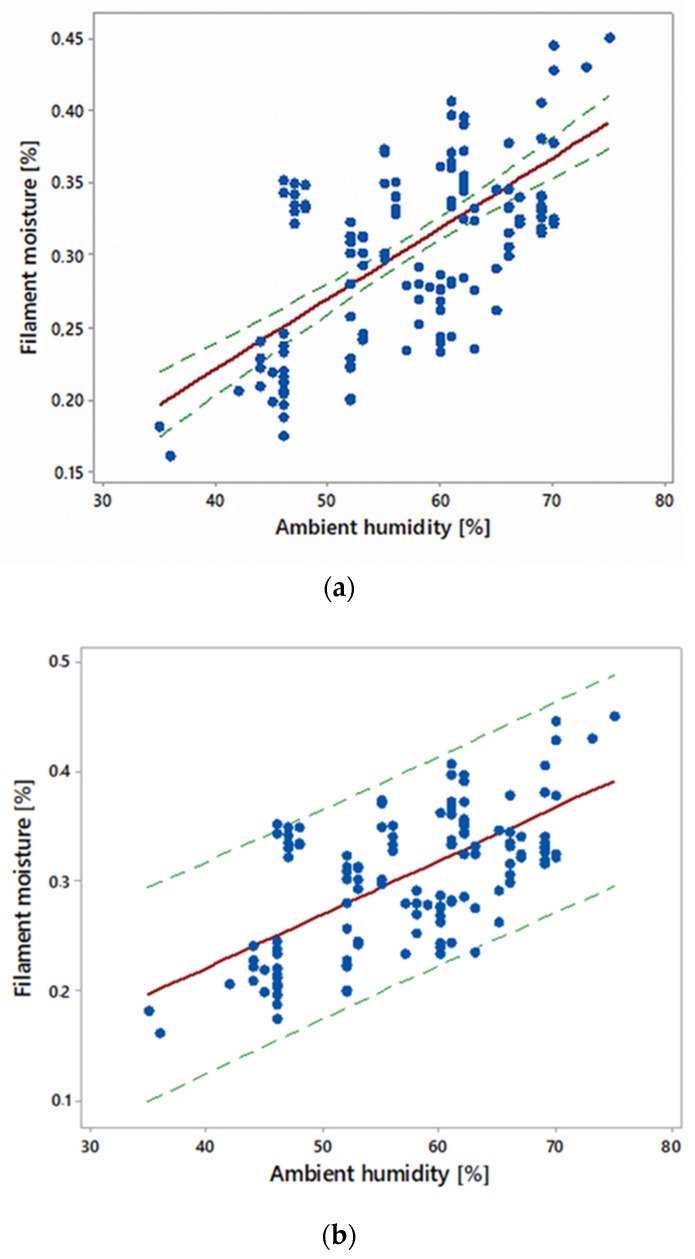
Regression line (red) between ambient humidity and filament moisture (sample size *n* = 150): (**a**) with confidence interval (green) for the mean; (**b**) with prediction interval (green) for a single observation; confidence level = 0.95.

Filament from all manufacturers was stored in spools and bundles in an environment with an ambient humidity of 35–75%. The air temperature changed during this period in the range of 21–23 °C and had an air pressure of 990–1020 hPa. The moisture measurements were carried out on 150 samples. The results are shown in Figure 6 in the form of a scatter plot, as well as a regression line and confidence interval for the mean (a) and for a single observation (b).

With an increase in the ambient humidity in the range of 35–75%, the moisture of the filament increased in the range of 0.15–0.45%. The regression function between these quantities is given by the following equation:

*FM* = 0.0049 *AH* + 0.0246 (2)

and the correlation coefficient was r = 0.64. The value of the correlation coefficient indicates that the significance of this relationship is relatively low [42]. From a practical point of view, however, this relationship is important and must be taken into account when planning the manufacturing process. It can be estimated that a change in the ambient humidity by 10 percentage points caused an increase in the filament humidity of 0.046%.

### 3.4. Mechanical Properties of the Filament Depending on Its Moisture

**Figure 7:**
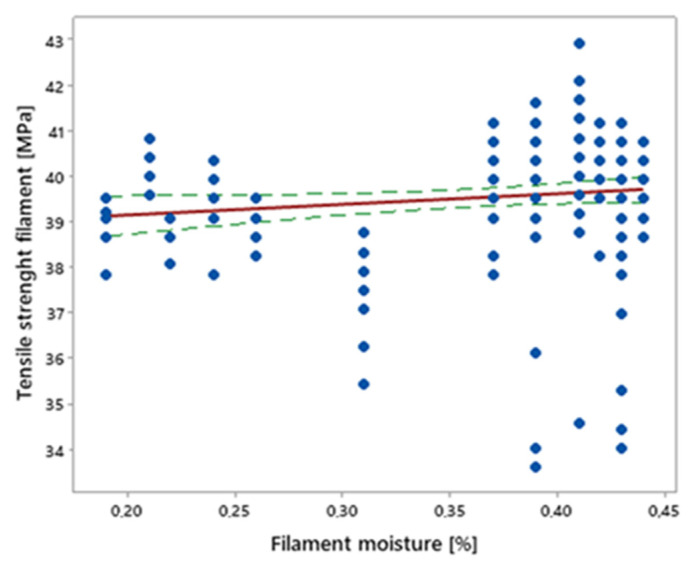
Influence of filament moisture on its tensile strength (red—regression line, green—confidence interval; confidence level = 0.95.

Filament samples with a moisture content in the range of 0.18–0.45% were subjected to tensile tests. The measurement results are shown in Figure 7 in the form of a regression line and the confidence interval of the mean.

The diagram shows that the tensile strength of the filament did not have any significant dependence on its moisture content—the correlation coefficient was 0.14. The strength spread, in the whole range of moisture variability, was equal to +/− 3 MPa.

### 3.5. Tensile Strength of the Printed Samples Depending on the Filament Moisture

From samples with moisture within the range shown in Section 3.4, printed samples were made using FFF. The printing conditions for the samples are presented in Table 3.

**Figure 8:**
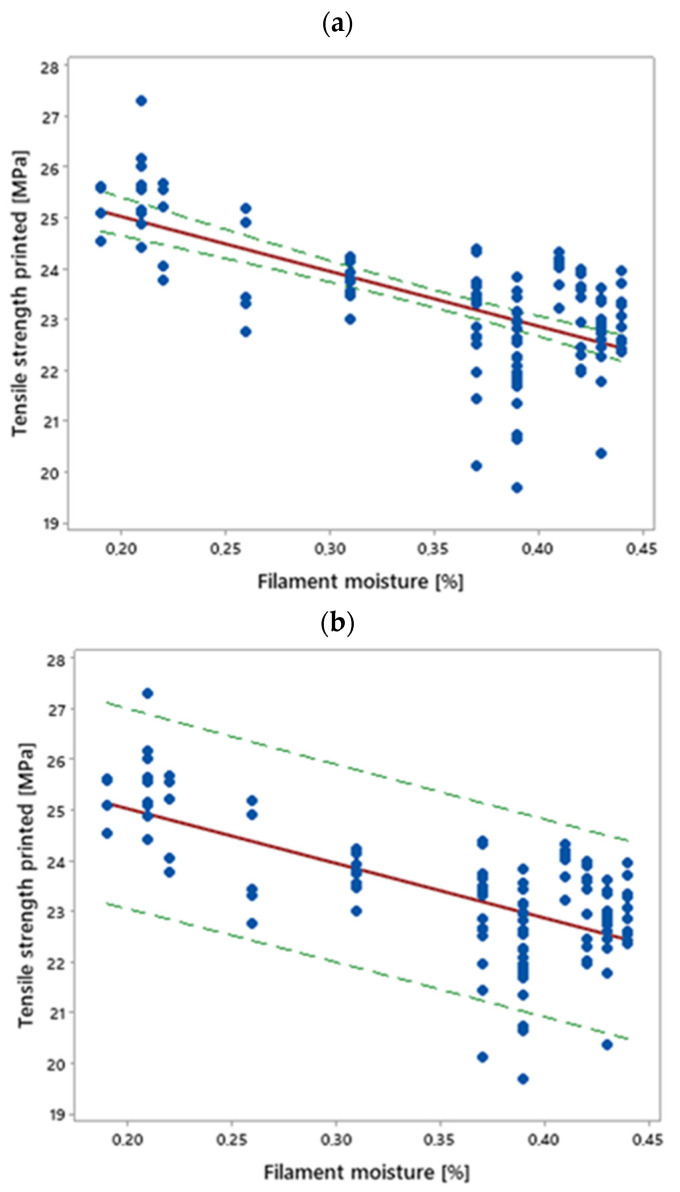
Regression line (red) between the tensile strength of the samples and the moisture of the filament (sample size *n* = 120): (**a**) with confidence interval (green) of the mean; (**b**) with prediction interval (green) for a single observation; confidence level = 0.95.

The results of measuring the tensile strength (*TS*) of the samples are shown in Figure 8a in the form of a scatter plot, a regression line, and a confidence interval, as a function of the filament moisture.

The results show that with an increase in the filament moisture content (*FM*), the tensile strength of the samples decreased. The regression function between these quantities is given by the equation:

*TS* = 10.80 *FM* − 27.19 (3)

and the correlation coefficient, r = −0.67, was quite low [42]. However, the dependence was significant from a practical point of view. It can be estimated that a change in the moisture of the filament by 0.1 percentage point caused a reduction in tensile strength by 1.08 MPa. Given Equation (2), it can be concluded that a change in the ambient humidity from 10 percentage points caused a reduction in the tensile strength of the sample by about 0.54 MPa.

### 3.6. Influence of Filament Moisture on the Surface Quality of Printed Samples

**Figure 9:**
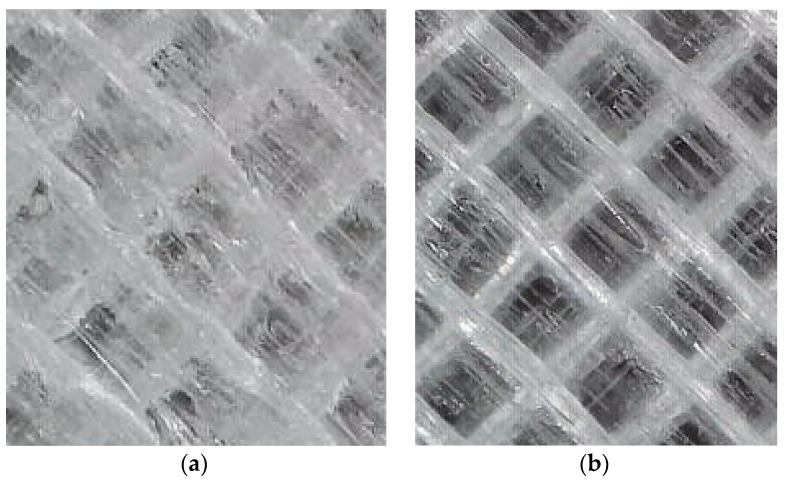
Influence of material moisture on the quality of the surface of the printed samples. (magnification 240×): (**a**) FM—0.41%; (**b**) FM—0.19%.

The filament humidity deteriorated the surface quality of the printed samples. The samples printed from a low-moisture filament had a homogeneous structure and low roughness. The samples printed from a high-moisture material were characterized by high surface roughness and clear traces of filament paths [31]. This is illustrated in Figure 9.

### 3.7. Changes in the Filament Moisture Depending on the Storage Method

The results of the research show that the ambient humidity significantly influenced the filament moisture, which in turn had a negative impact on the strength, and above all, on the quality of the surface of the prints. Guided by this finding, research was carried out on changes in the moisture content of the ABS filament stored in:

− The original packaging;
− A vacuum container;
− A container with a desiccant.

**Figure 10:**
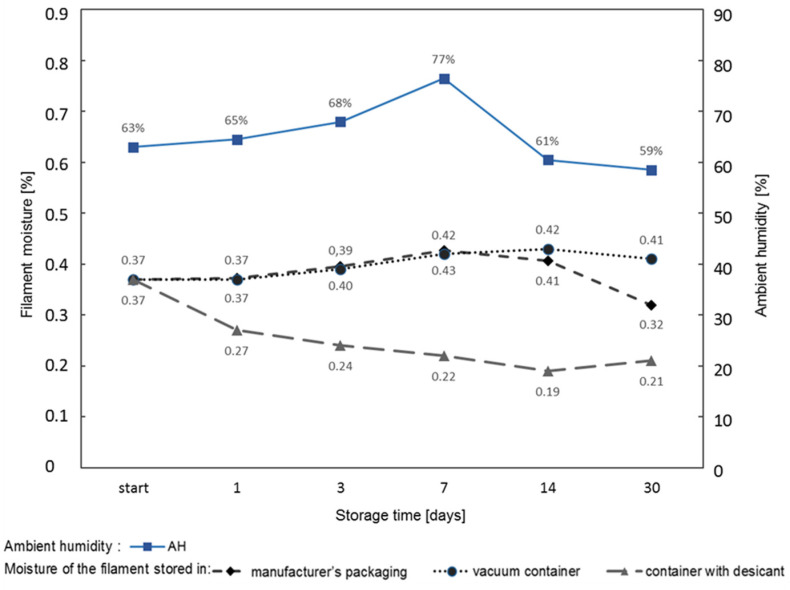
Changes in the moisture of the filament stored in various environments.

The results of the measurements are shown in Figure 10. In each case, the starting filament moisture was 0.37%. The research was carried out over a period of 30 days. During this period, the ambient humidity was 59–77%. Analysis of the chart in Figure 10 shows:

- The moisture of the filament stored in the original packaging varied in the range of 0.37–0.43%;
- The moisture of the filament stored in a vacuum container, in which the initial air humidity was 64%, varied in the range of 0.37–0.42%. The decrease in the humidity in the container and the decrease in the moisture of the filament after 30 days were most likely due to container leakage. As the air humidity in the container dropped to 59% after 30 days, drier air flowed into the container, which resulted in a decrease in the filament moisture;
- The moisture of the filament stored in the desiccant container decreased from an initial value of 0.37% to a value of 0.19%, and then it started to increase. This means that at a certain saturation state, the absorber stopped absorbing moisture, and even returned it to the filament stored in the container. It follows that the amount of absorber should be calculated based on the length of the filament storage time.

## 4. Conclusions

In this paper, it has been shown that the moisture of a filament made of ABS material changed proportionally to the humidity of the environment in which it was stored. Within the range of natural air humidity of 35–75%, with an ambient temperature of 21–23 °C, the moisture of the filament varied in the interval of 0.15–0.5%. This range of moisture corresponded to the natural moisture of the filament defined in the title of the article.

Considering that the given limits depend on factors that were not taken into account in the presented tests (see Equation (1)), the following ranges of natural filament moisture can be assumed:

− Residual humidity—up to 0.1%;
− Natural humidity—0.1–0.6%;
− Special humidity—over 0.6%.

These values are largely consistent with those given in the literature on the subject.

The results show that an increase in the ambient humidity by 10%, within the range of 35–75%, resulted in an average linear increase in the natural filament moisture by 0.049% (see Equation (1)).

Within the range of the natural humidity of the filament, the moisture content had little effect on its strength properties. However, it had a significant impact on the strength and surface quality of the prints made from the filament. The research presented in the article shows that this effect, in the filament moisture range of 0.18–0.44%, can be described by a linear relationship, with a proportionality coefficient of 10.80, which means that a change in the filament humidity by 0.1% resulted in a 1.08 MPa change in tensile strength. 

The influence of the ambient humidity on the moisture of the filament, and the moisture of the filament on the strength and quality of the surface of the printed parts, was significant from a practical point of view. However, it should be emphasized that the obtained relationships were statistically relatively weak. The correlation coefficients of these values were r = 0.64 and −0.67, respectively. This can be explained by the heterogeneity of the research sample. The materials used in the research were delivered by several manufacturers, and the storage times of the filaments in specific humidity levels of the environment were different. Obtaining such a heterogeneous sample was, however, intentional, because the aim of the research was to determine the relationship in the widest possible range of variability in the external conditions.

In future research, it will be necessary to examine the impact of other environmental factors, primarily the ambient temperature and the exposure time, on the humidity absorption of the filament.

## Figures and Tables

**Table 1:** Summary of literature review.

		Material	Range of Filament Moisture (%)	The Range of Changes in Mechanical Properties of Printed Samples
[9]	Valerga A.P., M. Batista et al.	PLA	lack of data	lack of data
[20]	Zaldivar R.J, Mclouth T.D. et al.	PEI	0.04–0.8	UTC = 20.7–56.8 MPa
[21]	Halidi S.N.A.M, Abdullah J.	ABS	0.45–1.1	lack of data
[22]	Wichniarek R. Hamrol A. et al.	ABS	0.1–0.6	UTC = 29.16–33.39 MPa
[25]	Kariz M, Sernek M. et al.	ABS	0.40–1.1	MOE =1313–1383 MPa
[25]	Kariz M, Sernek M et al.	PLA	0.3–1.8	MOE = 1477–1568 MPa
[30]	Janas S. Kwiecień I. Kowalska M.	ABS	0.10–1.8	lack of data

**Table 2:** The properties of the ABS material used in the research [39].

Properties	Testing Method	Unit	Typical Value
Physical properties			
Density	ISO 1183	g/cm^3^	1.04
Water absorption	24 h, 20 °C	%	0.7
Moisture absorption	24 h, 20 °C	%	0.2
Mechanical properties			
Tensile modulus of elasticity	ISO 527	MPa	1750
Tensile strength yield	ISO 527	MPa	42
Elongation yield	ISO 527	%	5%
Thermal properties			
Glass transition temperature	DSC	°C	105
Softening point	ISO 306	°C	100

**Table 3:** Conditions for printed samples.

Printer	
Type	Zotrax M200 Plus (with Closed Printing Chamber)
Printing parameters	
Extrusion temperature	275 °C
Temperature of the working plate	80 °C
Layer thickness	0.19 mm
Nozzle diameter	0.4 mm
Sample	
Degree of filling	100%
Ambient condition	
Ambient humidity	45–50%
Ambient temperature	19–21 °C

## Data Availability

There is no additional data.
